# Quantifying and Valuing Community Health Worker Time in Improving Access to Malaria Diagnosis and Treatment

**DOI:** 10.1093/cid/ciw629

**Published:** 2016-12-06

**Authors:** Joëlle Castellani, Borislava Mihaylova, IkeOluwapo O. Ajayi, Mohamadou Siribié, Jesca Nsungwa-Sabiiti, Chinenye Afonne, Luc Sermé, Andrew Balyeku, Vanessa Kabarungi, Josephine Kyaligonza, Silvia M. A. A. Evers, Aggie T. G. Paulus, Max Petzold, Jan Singlovic, Melba Gomes

**Affiliations:** 1 Department of Health Services Research, School for Public Health and Primary Care (CAPHRI), Maastricht University, The Netherlands; 2 Health Economics Research Centre, Nuffield Department of Population Health, University of Oxford, United Kingdom; 3 Department of Epidemiology and Medical Statistics, College of Medicine, University of Ibadan, Nigeria; 4 Groupe de Recherche Action en Santé, Ouagadougou, Burkina Faso; 5 Child Health Division, Ministry of Health, Kampala, Uganda; 6 Epidemiology and Biostatistics Research Unit, Institute of Advanced Medical Research and Training, College of Medicine, University of Ibadan, Nigeria; 7 Centre for Applied Biostatistics, Occupational and Environmental Medicine, Sahlgrenska Academy, University of Gothenburg, Sweden; 8 UNICEF/UNDP/World Bank/WHO/Special Programme for Research & Training in Tropical Diseases, World Health Organization, Geneva, Switzerland

**Keywords:** CHW, opportunity cost, workload, ACTs, RDTs

## Abstract

***Background.*** Community health workers (CHWs) are members of a community who are chosen by their communities as first-line, volunteer health workers. The time they spend providing healthcare and the value of this time are often not evaluated. Our aim was to quantify the time CHWs spent on providing healthcare before and during the implementation of an integrated program of diagnosis and treatment of febrile illness in 3 African countries.

***Methods.*** In Burkina Faso, Nigeria, and Uganda, CHWs were trained to assess and manage febrile patients in keeping with Integrated Management of Childhood Illness recommendations to use rapid diagnostic tests, artemisinin-based combination therapy, and rectal artesunate for malaria treatment. All CHWs provided healthcare only to young children usually <5 years of age, and hence daily time allocation of their time to child healthcare was documented for 1 day (in the high malaria season) before the intervention and at several time points following the implementation of the intervention. Time spent in providing child healthcare was valued in earnings of persons with similar experience.

***Results.*** During the high malaria season of the intervention, CHWs spent nearly 50 minutes more in daily healthcare provision (average daily time, 30.2 minutes before the intervention vs 79.5 minutes during the intervention; test for difference in means *P* < .01). On average, the daily time spent providing healthcare during the intervention was 55.8 minutes (Burkina Faso), 77.4 minutes (Nigeria), and 72.2 minutes (Uganda). Using the country minimum monthly salary, CHWs’ time allocated to child healthcare for 1 year was valued at US Dollars (USD) $52 in Burkina Faso, USD $295 in Nigeria, and USD $141 in Uganda.

***Conclusions.*** CHWs spend up to an hour and a half daily on child healthcare in their communities. These data are informative in designing reward systems to motivate CHWs to continue providing good-quality services.

***Clinical Trials Registration.*** ISRCTN13858170.

The run-up to meet the Millennium Development Goals witnessed an upsurge in programs using community health workers (CHWs) to improve health, particularly child health [1]. CHWs offer a wide range of services to their communities in low-income countries and improve access to healthcare by reaching the poorest and most inaccessible areas [2]. Because health facilities often concentrate on curative care over prevention, and families in poorer areas are less likely to reach health facilities than those in wealthier areas [3], CHWs are seen as a means of bringing both preventive and curative care closer to the patient, improving health and reducing inequities. CHWs perform a wide range of tasks, from first aid and treatment of simple, common illnesses to interventions that promote healthy behavior; some of them also keep records and collect data on vital events [2]. In their case management roles, CHWs do not replace the need for skilled health workers, but when they are trained and assigned to manage simple tasks in their communities, including screening for illness condition and referring patients who need specialized management, and when properly supervised and supported, they become effective links to the formal healthcare system [4]. Several studies have shown a decrease of neonatal and child mortality when early signs of severe illness are identified and interventions delivered by CHWs in the communities [5–8].

In sub-Saharan Africa, CHWs' activities are largely voluntary and unpaid. However, the evidence on the extent to which CHWs can be effective in improving health has led to an increase in the number of tasks they are assigned without financial remuneration, while their performance is increasingly examined, which has raised stress and increased attrition [2, 9]. The time CHWs contribute to their community reduces the time they can allocate to other tasks, including care of their own children and, as their healthcare activities are voluntary, their income may also decrease. However, assessments of the volume of activities CHWs provide, the time allocated to these tasks, and whether and how this time load affects other tasks and income are extremely rare [10, 11].

Given the number of health programs that depend upon CHWs to implement health interventions in their communities without quantifying the impact upon their time, our goal was to redress the balance. We aimed to quantify changes in time allocation that were brought by an intervention using CHWs to increase access to diagnosis and treatment for malaria in remote communities in 3 African countries. The intervention involved the provision in malaria-endemic communities of malaria rapid diagnostic tests (RDTs), and for malaria-positive cases, oral artemisinin-based combination therapies (ACTs), or for patients who need immediate transit to health facilities, pre-referral rectal artesunate [12].

## METHODS

### Study Sites

This time-allocation study was carried out in 42 villages before the intervention and in 124 villages during the intervention in 4 rural malaria-endemic areas of Burkina Faso (health area of Sidéradougou, Health District of Mangodara), Nigeria (Ona-Ara local government area), and Uganda (Kayunga and Sheema districts). The aim was to quantify the number of hours of time spent by CHWs on different activities during the course of a normal day before and following the implementation of an intervention delivered by CHWs to diagnose and treat malaria.

Data collection for the pre-intervention phase of the time-allocation study took place between May and September 2013 in Nigeria and during the month of August 2014 in Burkina Faso; for the intervention phase, time-allocation data were collected between January 2014 and October 2015 in Nigeria, between January and July 2015 in Uganda, and between May and September 2015 in Burkina Faso. There was no pre-intervention time-allocation study in Uganda because there were no CHWs operating before the intervention. In Nigeria, those functioning as CHWs previously (some participating in previous studies) and those selected for the intervention were interviewed. Nineteen study communities in Burkina Faso and 23 in Nigeria before the intervention and 32 study communities in Burkina Faso, 14 in Nigeria, and 78 in Uganda during the intervention participated in this time-allocation study.

### Role of the CHWs

In all 3 countries, health centers are situated far away from people's homes, and travel to health centers is expensive. Therefore, CHWs, typically chosen by their communities, are often an initial point of healthcare treatment before referral to the next level of care. The intervention involved training CHWs to assess, diagnose, treat, and refer children in their communities according to standard treatment guidelines [13]. Once successfully identified and trained, the CHWs were provided with malaria RDTs, ACTs, and rectal artesunate, and supported in their diagnosis and treatment of RDT-positive patients, identification of danger signs (repeated vomiting, unable to eat/drink/suck, convulsions, altered consciousness, lethargy, and difficulties in breathing) and provision of rectal artesunate to patients with danger signs before referring them to the nearest health facility for further management [12].

In each country, CHWs voluntarily contribute to health services, and this practice did not change during the study. In Burkina Faso, as per government policy, ACTs are subsidized by the Ministry of Health and CHWs are permitted to retail subsidized ACTs at 100 West African CFA francs (XOF) (US Dollars [USD] $.17) and 200 XOF (USD $.33) for children younger and older than 37 months respectively. In Nigeria, CHWs were unpaid but received gifts during festivities and gifts if they performed well (number of cases, quality of record-keeping), and were reimbursed for transport to meetings. They were paid USD $10 per month in Nigeria and USD $3 per visit in Uganda for their transport to replenish commodities. Short message service (SMS)/phone calls regarding specific patients were reimbursed. A stipend was given for team meeting days. Conforming to policy in Nigeria and Uganda, RDTs and ACTs were provided at no cost to patients; in all countries, rectal artesunate was provided at no cost to the patient.

### Questionnaire Design and Data Collection

Basic characteristics of the CHWs such as gender, age, marital status, occupation, and number of years of experience as a CHW were recorded before and during the intervention.

During the intervention, any use of RDTs and medicines was recorded by the CHWs. The CHWs monitored their allocation of time during the day, but also measured for a random sample of treated children the time needed for the guardians to give an illness history; the time used to prepare, perform, and read a RDT; the time spent waiting for results and giving explanations of the RDT procedure; the time for providing medicine to a child; and the time needed to explain the referral and follow-up process. Information on test results, severity of the episode with or without any danger signs (difficult/fast breathing, repeated vomiting, unable to eat/drink/suck, pallor, convulsions/chills/rigors, weakness/lethargy, anorexia, bulging fontanel, coma/altered consciousness), treatments given, and referral was recorded. The children for whom the CHW monitored time allocation in greater detail were randomly selected, and data were usually obtained on the same day as other data for daily time allocation were collected.

Structured case report forms (CRFs) were in French for Burkina Faso and in English for Nigeria and Uganda. Each CHW was asked to list what they did on the day before, from 6 am to 6 pm, in slots of 20 minutes. Activities listed by the CHWs consisted of healthcare provision, family/personal time and housework (eg, cleaning the house, looking after the children, washing the clothes/dishes), agriculture, paid work (eg, teaching), self-employment (eg, tailor), and going to the market. Completing forms took approximately 30 minutes to 1 hour. Local translations (except for Burkina Faso) were made and tested before use. In Burkina Faso and in Nigeria, during the pre-intervention data collection, each CHW was visited once by an investigator who filled in the CRF for the CHW. During the intervention, CHWs were trained to fill in the questionnaires themselves, except in Uganda where a trained research assistant (11 research assistants in Sheema and 10 in Kayunga) helped CHWs to fill the forms.

### Data Analysis

The activities that contributed to the CHWs' healthcare provision were detailed (taking care of sick children, working as health workers, going to CHW meetings, etc). For each completed CRF, time in different activities was aggregated and mean time (in minutes) reported. Time for guardians to give the history of the illness, for preparation and performing a RDT, waiting for results, explaining RDT procedures, treating the child, and explaining the referral and follow-up process was quantified.

Only CHWs were included. Formal health sector staff (n = 2 before intervention) and owners of drug shops (n = 7 before intervention; n = 5 during intervention) were excluded. A maximum number of CRFs per CHW was 8 forms irrespective of the number completed (varied numbers were completed: 9–59 in Nigeria during the intervention phase and 9–58 for Burkina Faso and Nigeria for the CRF on the time needed to perform RDTs and give treatment). All forms were filled within 24 hours of respective activities to reduce risks of recall bias.

In Burkina Faso, CHWs charged patients for each treatment pack. We calculated income received from ACTs using treatment data from registers, numbers of children assessed, child's age, and treatment received. These data were used to calculate the average income from retailing ACTs per month during low and high malaria seasons, as well as the annual income.

The CHWs’ time for healthcare was valued using external data on minimal monthly salary in each country. In Burkina Faso, the hourly minimum wage was 176.83 XOF (about 28 292 XOF per month or USD $52.10) [14]. In Nigeria, the monthly minimum wage was 18 000 Nigerian naira (NGN) (USD $100) [14] and in Uganda, USD $54 [11]. CHWs’ time value is presented in US dollars (USD $) using average exchange rates between 2014 and 2015: USD $1 = 542.75 XOF (Burkina Faso) and USD $1 = 180 NGN (www.oanda.com).

### Statistical Methods

Data were double entered, separately for each country, in EpiData 3.1 and analyzed using Stata software version 13.0 (StataCorp, College Station, Texas). We report average daily times as well as distribution of time in the following categories: none, >0–2 hours, >2 hours; or none, >0–4 hours, >4–8 hours, and >8 hours. Two-tailed *t* tests were used to compare means and χ^2^ tests for heterogeneity to compare distributions of times allocated to a particular activity before and during the intervention in each country and across countries. *P* values <.05 indicated statistically significant differences.

### Ethics Issues

The research protocol of the main study was approved by the National Health Research Committee, the University of Ibadan/University College Hospital Institutional Review Committee, and the Oyo State Ministry of Health in Nigeria; the National Ethics Committee for the Research on Health and the National Regulatory Authority in Burkina Faso; the National Council for Science and Technology in Uganda; and the World Health Organization Research Ethics Review Committee. In each country, CHWs as well as guardians of sick children gave consent for the main study.

## RESULTS

### Exclusion of CHWs for This Sub-research

During the pre-intervention phase, in Burkina Faso, 3 CHWs who filled the forms were excluded because 1 was a minor at the time of the study, and 2 did not take part in the intervention because 1 was not accepted by the community and the other was not able to calculate the age of the children.

### Profile of the CHWs Contributing to the Time-Allocation Study

In Burkina Faso and Nigeria, 20 and 42 CHWs, respectively, took part in the pre-intervention phase of the time-allocation study; there were no CHWs yet in the study area of Uganda (Table 1). During the intervention, 36 CHWs in Burkina Faso, 17 CHWs in Nigeria, and 147 CHWs in Uganda participated. In Nigeria, for cultural reasons, all CHWs were females while in Burkina Faso, a larger proportion of CHWs were males (75%). In Uganda, about 75% of CHWs were females.

**Table 1.** Characteristics of Community Health Workers

Category	Before Intervention		During Intervention		
	Burkina Faso	Nigeria	Burkina Faso	Nigeria	Uganda
No. of community health workers	20	42	36	17	147
Gender					
Male	15 (75)	…	27 (75)	…	37 (25)
Female	5 (25)	42 (100)	9 (25)	17 (100)	110 (75)
Age, y					
≤20	…	…	1 (3)	…	…
21–30	9 (45)	8 (19)	13 (36)	1 (6)	25 (17)
31–40	4 (20)	14 (33)	10 (28)	6 (36)	62 (42)
41–50	6 (30)	10 (24)	11 (30)	5 (29)	42 (29)
>50	1 (5)	10 (24)	1 (3)	5 (29)	16 (11)
Missing data	…	…	…	…	2 (1)
Marital status					
Single	1 (5)	…	3 (8)	…	5 (3)
Married	19 (95)	37 (88)	32 (89)	13 (76)	129 (88)
Separated	…	2 (5)	…	…	…
Divorced	…	…	…	2 (12)	5 (3)
Widowed	…	3 (7)	1 (3)	2 (12)	8 (6)
Main occupation					
Only farming	14 (70)	4 (10)	32 (89)	1 (6)	111 (76)
Paid employment mainly	2 (10)	6 (14)	…	3 (18)	11 (7)
Self-employment mainly	4 (20)	32 (76)	3 (8)	13 (76)	22 (15)
Housewife/unemployed	…	…	1 (3)	…	3 (2)
How long served as a community health worker					
<1 y	4 (20)	22 (52)	10 (28)	…	9 (6)
1 y to <2 y	2 (10)	2 (5)	…	2 (12)	15 (10)
2 y to <3 y	…	…	4 (11)	2 (12)	34 (23)
≥3 y	14 (70)	18 (43)	22 (61)	13 (76)	89 (61)

Data are presented as No. (column %).

In all 3 countries, most CHWs were between 21 and 50 years old (>70%) and mainly married (>75%). Their main occupation was farming in Burkina Faso (>70%) and in Uganda (76%) but mainly self-employed in Nigeria (76%). The majority of CHWs had been working as CHWs for at least 2 years (>70%). During the pre-intervention phase, Nigerian CHWs were not officially in place but had participated in previous studies on community management of malaria; more than half (57%) had <2 years of experience while the remaining CHWs (43%) had at least 3 years of experience.

### Time Allocation of the CHWs

Data on time spent on categories of daily activities during the high malaria season (ie, pre-intervention questionnaires were completed exclusively during the high malaria season) are summarized in Table 2. Before the intervention, the average daily time spent by CHWs on healthcare activities was 30.16 minutes. After the provision of training and commodities (RDTs and medicines), CHWs spent on average 79.47 minutes (test for difference in mean times before vs during the intervention, *P* < .01, with effects similar separately among sites in Burkina Faso and Nigeria). However, although their average daily time spent on family activities appears increased (246.23 before vs 325.79 during intervention; *P* < .01), their time spent on self-employment (166.56 vs 30.79 minutes; *P* < .0001) had decreased. The effect on time spent on self-employment was driven by a large effect observed exclusively in Nigeria (246.83 vs 23.03 minutes; *P* < .0001). There were no important differences in mean daily time spent on agricultural activities or paid work (*P* values not significant).

**Table 2.** Time Allocation for Different Daily Activities During the High Malaria Season^a^

Daily Activity	Before Intervention			During Intervention		
	Burkina Faso (20 CHWs)	Nigeria (41 CHWs)	Total (61 CHWs)	Burkina Faso (33 CHWs)	Nigeria (13 CHWs)	Total (46 CHWs)
No. of CHW questionnaires	20	41	61	43	33	76
Healthcare provision						
0 h	17 (85)	38 (93)	55 (90)	5 (12)	1 (3)	6 (8)
>0–2 h	1 (5)	2 (5)	3 (5)	30 (70)	30 (91)	60 (79)
>2 h	2 (10)	1 (2)	3 (5)	8 (18)	2 (6)	10 (13)
Mean time, min (SD)	53.00 (160.95)	19.02 (101.19)	30.16 (123.65)	89.77 (63.00)	66.06 (36.57)	79.47 (54.16)
Test for heterogeneity in distributions	…	…	…	*P* < .0001	*P* < .0001	*P* < .0001
Test for difference in means	…	…	…	*P* = .3274	*P* = .0073	*P* = .0044
Family/personal time and housework^b^						
0 h	1 (5)	1 (2)	2 (3)	1 (2)	…	1 (1)
>0–4 h	12 (60)	19 (46)	31 (51)	18 (42)	6 (18)	24 (32)
>4–8 h	6 (30)	19 (46)	25 (41)	21 (49)	14 (42)	35 (46)
>8 h	1 (5)	2 (5)	3 (5)	3 (7)	13 (39)	16 (21)
Mean time, min (SD)	218.00 (114.78)	260.00 (133.64)	246.23 (128.35)	247.91 (167.79)	427.27 (175.91)	325.79 (192.30)
Test for heterogeneity in distributions	…	…	…	*P* = .4816	*P* = .0015	*P* = .0164
Test for difference in means	…	…	…	*P* = .4123	*P* < .0001	*P* = .0044
Agriculture						
0 h	1 (5)	24 (59)	25 (41)	5 (12)	13 (39)	18 (24)
>0–4 h	2 (10)	11 (27)	13 (21)	9 (21)	8 (24)	17 (22)
>4–8 h	9 (45)	5 (12)	14 (23)	19 (44)	11 (33)	30 (39)
>8 h	8 (40)	1 (2)	9 (15)	10 (23)	1 (3)	11 (14)
Mean time, min (SD)	420.00 (185.36)	99.51 (147.34)	204.59 (219.92)	323.72 (189.30)	169.09 (177.84)	256.58 (198.78)
Test for heterogeneity in distributions	…	…	…	*P* = .4065	*P* = .1584	*P* = .1058
Test for difference in means	…	…	…	*P* = .0612	*P* = .0753	*P* = .1536
Paid work						
0 h	20 (100)	38 (93)	58 (95)	42 (98)	32 (97)	74 (97)
>0–4 h	…	…	…	…	1 (3)	1 (1)
>4–8 h	…	3 (7)	3 (5)	1 (2)	…	1 (1)
Mean time, min (SD)	0.00 (0.00)	28.78 (104.12)	19.34 (86.10)	6.05 (39.65)	7.27 (41.78)	6.58 (40.31)
Test for heterogeneity in distributions	…	…	…	*P* = .9254	*P* = .4064	*P* = .3907
Test for difference in means	…	…	…	*P* = .3209	*P* = .2311	*P* = .2877
Self-employment						
0 h	19 (95)	15 (36)	34 (56)	37 (86)	26 (79)	63 (83)
>0–4 h	1 (5)	6 (15)	7 (11)	3 (7)	7 (21)	10 (13)
>4–8 h	…	13 (32)	13 (21)	2 (5)	…	2 (3)
>8 h	…	7 (17)	7 (11)	1 (2)	…	1 (1)
Mean time, min (SD)	2.00 (8.94)	246.83 (231.77)	166.56 (221.95)	36.74 (107.17)	23.03 (47.47)	30.79 (86.25)
Test for heterogeneity in distributions	…	…	…	*P* = .8849	*P* = .0037	*P* = .0001
Test for difference in means	…	…	…	*P* = .0388	*P* < .0001	*P* < .0001
Going to the market						
0 h	20 (100)	30 (73)	50 (82)	39 (91)	23 (70)	62 (82)
>0–2 h	…	4 (10)	4 (7)	1 (2)	9 (27)	10 (13)
>2 h	…	7 (17)	7 (11)	3 (7)	1 (3)	4 (5)
Mean time, min (SD)	0.00 (0.00)	65.37 (141.07)	43.93 (119.27)	13.95 (49.48)	26.67 (46.55)	19.47 (48.33)
Test for heterogeneity in distributions	…	…	…	*P* = .6988	*P* = .0376	*P* = .2155
Test for difference in means	…	…	…	*P* = .0692	*P* = .1777	*P* = .1345

Data are presented as No. (column %) unless otherwise indicated. Percentages are rounded and sometimes do not add to 100%.

Abbreviations: CHW, community health worker; SD, standard deviation.

^a^ High malaria season: Burkina Faso: July, August, and September; Nigeria: January, May, June, July, and August.

^b^ Includes taking care of their own children, cleaning the house, lighting a fire, cooking, fetching water, washing the clothes/dishes, taking a bath, eating, having lessons, praying/going to church, visiting someone, taking a nap.

When all data of CHWs' time allocation during the intervention (ie, not just during high malaria season) are taken into account, the time spent on healthcare activities was on average 55.8 minutes in Burkina Faso, 77.4 minutes in Nigeria, and 72.2 minutes in Uganda (Table 3). Most of the daily time was allocated to agriculture in Burkina Faso (345.6 minutes) and to family/personal time in Nigeria and Uganda (395.1 minutes for Nigeria and 334.9 minutes in Uganda). When the time was stratified by low and high malaria seasons, more time on healthcare activities was spent during the high malaria season in Burkina Faso and in Uganda (Supplementary Table 1). As a consequence, family/personal time as well as the time spent on agriculture decreased. However, in Nigeria, CHWs spent on average 21.5 minutes less time providing healthcare during the high malaria season compared with the low malaria season.

**Table 3.** Allocation of Community Health Workers’ Time Across Categories of Daily Activities During the Intervention Period

Daily Activity	During Intervention		
	Burkina Faso (36 CHWs)	Nigeria (17 CHWs)	Uganda (147 CHWs)
No. of CHW questionnaires	72	70	467
Healthcare provision			
0 h	31 (43)	3 (4)	26 (6)
>0–2 h	33 (46)	61 (87)	411 (88)
>2 h	8 (11)	6 (9)	30 (6)
Mean time, min (SD)	55.8 (64.9)	77.4 (65.0)	72.2 (55.9)
Family/personal time and housework^a^			
0 h	1 (1)	…	17 (4)
>0–4 h	34 (47)	14 (20)	101 (21)
>4–8 h	31 (43)	36 (51)	299 (64)
>8 h	6 (8)	20 (29)	50 (11)
Mean time, min (SD)	254.4 (162.2)	395.1 (166.4)	334.9 (141.7)
Agriculture			
0 h	7 (10)	28 (40)	35 (7)
>0–4 h	15 (21)	22 (32)	186 (40)
>4–8 h	29 (40)	17 (24)	231 (50)
>8 h	21 (29)	3 (4)	15 (3)
Mean time, min (SD)	345.6 (194.7)	156.9 (172.0)	257.7 (121.6)
Paid work			
0 h	71 (99)	66 (94)	464 (99)
>0–4 h	…	2 (3)	1 (0)
>4 h	1 (1)	2 (3)	2 (1)
Mean time, min (SD)	3.6 (30.6)	16.6 (69.5)	2.3 (32.1)
Self-employment			
0 h	61 (85)	45 (64)	411 (88)
>0–4 h	4 (6)	23 (33)	21 (5)
>4–8 h	5 (7)	2 (3)	25 (5)
>8 h	2 (3)	0 (0)	10 (2)
Mean time, min (SD)	48.3 (130.0)	44.3 (82.5)	39.7 (124.4)
Going to the market			
0 h	67 (93)	42 (60)	403 (86)
>0–2 h	1 (1)	27 (39)	54 (12)
>2 h	4 (6)	1 (1)	10 (2)
Mean time, min (SD)	10.6 (42.6)	29.1 (41.8)	13.2 (38.9)

Data are presented as No. (column %) unless otherwise indicated. Percentages are rounded and sometimes do not add to 100%. Data of all participating CHWs are included.

Abbreviations: CHW, community health worker; SD, standard deviation.

^a^ Includes taking care of their own children, cleaning the house, lighting a fire, cooking, fetching water, washing the clothes/dishes, taking a bath, eating, having lessons, praying/going to church, visiting someone, taking a nap.

We evaluated the average number of hours spent by CHWs per year in providing healthcare in the communities to 159.5 in Burkina Faso, 471.9 in Nigeria, and 417.0 in Uganda. With an average salary per month (US dollars) of USD $52.1 (Burkina Faso), USD $100.0 (Nigeria), and USD $54.0 (Uganda), the value of the time that CHWs spent on treating sick children in the study areas was estimated at USD $2544 (Burkina Faso), USD $15 040 (Nigeria), and USD $23 081 (Uganda) per year (Supplementary Table 2).

### Use of Rapid Diagnostic Tests and Treatment Package

Table 4 presents the CHWs’ time per child for administering a RDT and appropriate treatment as well as the RDT results, the illness severity, and the treatment given during the intervention. In Uganda, a CHW needed on average 45.7 minutes, while the time needed in Burkina Faso and Nigeria was shorter (39.9 minutes in Burkina Faso and 32.1 in Nigeria). Most of the time needed concerned the preparation and the use of a RDT (18.0 minutes for Burkina Faso, 17.9 for Nigeria, and 14.5 minutes for Uganda). Most of the children were tested malaria positive and uncomplicated (>73%). Only a few of them had danger signs (<2.5%). Among the children included in the detailed time-allocation sub-study, >76% received ACT and only 2 received rectal artesunate (1 in Nigeria [0.9%] and 1 in Uganda [0.2%]), while about 23.6% of the children in Burkina Faso, 16.0% in Nigeria, and 13.8% in Uganda received no treatment or only paracetamol (acetaminophen). In Burkina Faso, because CHWs were allowed to retail ACTs to patients and could keep the profit margin for themselves, we estimated their income from ACTs to be USD $3.41 for the 3 months of high malaria season and USD $3.60 for the 9 months of low malaria season (Supplementary Table 3).

**Table 4.** Community Health Workers’ Time for Rapid Diagnostic Tests (RDT) and Treatment, Illness Severity, RDT Results, and Treatment Given

Characteristic	During Intervention					
	Burkina Faso		Nigeria		Uganda	
	No.	Mean (SD) or %	No.	Mean (SD) or %	No.	Mean (SD) or %
No. of febrile cases in children <5 years of age	195		106		465	
No. of community health workers	30		22		147	
Time used for RDTs and treatment, min, mean (SD)						
History of the illness	195	7.0 (4.4)	106	5.5 (3.1)	465	8.8 (7.3)
Preparation and taking a RDT	195	18.0 (7.2)	106	17.9 (5.5)	465	14.5 (8.1)
Giving results and explanations	195	5.3 (3.7)	106	4.2 (2.2)	465	10.6 (7.3)
Treatment	176	5.8 (3.7)	100	4.7 (1.9)	465	7.5 (5.0)
Referral and follow-up advice	181	4.9 (3.2)	2	5.0 (0.0)	373	5.4 (3.8)
Total time per RDT and treatment	195	39.9 (13.1)	106	32.1 (7.5)	465	45.7 (15.7)
RDTs results, severity, and treatment, No. (%)						
Uncomplicated febrile children^a^						
Malaria positive	144	73.9	81	76.4	389	83.7
Malaria negative	41	21.0	4	3.8	69	14.8
Not conclusive	0	0.0	1	0.9	0	0.0
Unable to take oral medications						
Malaria positive	7	3.6	3	2.8	5	1.1
Malaria negative	3	1.5	0	0.0	1	0.2
Missing severity						
Malaria negative	0	0.0	17	16.1	1	0.2
Treatment decisions						
ACT treatment provided	149	76.4	82	77.4	396	85.2
Rectal artesunate treatment provided	0	0.0	0	0.0	1	0.2
Rectal artesunate and ACT	0	0.0	1	0.9	0	0.0
Paracetamol (acetaminophin) + other drugs (recommended to be purchased by patient	21	10.8	15	14.1	0	0.0
No treatment given, but eventually recommendation to purchase other drugs	25	12.8	2	1.9	64	13.8
Given other treatment: paracetamol, antibiotics, Septrin, folic acid, ORS, amodiaquine, sulfadoxine/pyrimethamine	0	0.0	0	0.0	4	0.8
Missing	0	0.0	6	5.7	0	0.0
No. of children with danger signs seen/treated^b^	4	2.1	2	1.9	1	0.2

Abbreviations: ACT, artemisinin-based combination therapy; ORS, oral rehydration solution; RDT, rapid diagnostic test; SD, standard deviation.

^a^ Children with no danger signs (difficult/fast breathing, repeated vomiting, unable to eat/drink/suck, pallor, convulsions/chills/rigors, weakness/child not playing/lethargy, anorexia, bulging fontanel, coma/altered consciousness).

^b^ Danger signs mentioned above.

## DISCUSSION

A total of 265 CHWs provided access to diagnosis and treatment to about 16 000 young children in 162 villages in 3 countries. The intervention increased access to diagnosis and treatment by 80%, reduced the number of children with signs of severe illness from 24.7% to 18.1%, and reduced illness duration from 3.7 to 3.5 days for uncomplicated episodes and from 4.2 to 3.7 days for severe episodes [12]. This study shows that because of the intervention, the total time on healthcare activities by the CHWs rose from about 30 minutes to about 80 minutes overall (from 53 to 90 minutes in Burkina Faso and from 19 to 66 minutes in Nigeria). The daily volume of time spent on childcare was modest both before and after the intervention, and the bulk of the time was spent on diagnosis of malaria, with a smaller fraction on treatment. Our findings are considerably less than the 4.8 hours per day found in Ghana, but comparable to findings in Uganda reporting about 40 minutes daily taken by CHWs to provide integrated care for common childhood illnesses [10, 11].

All of the CHWs were contributing voluntarily. They had on average >3 years of experience, and most went to primary school in Burkina Faso and Nigeria and to secondary school in Uganda. Using the average income in each country for people of equivalent education and experience, the estimated value of their time during 1 year ranged from USD $51.9 in Burkina Faso to USD $294.9 in Nigeria. Some CHWs, but not all, benefited from payments in kind; most stated gaining improved community status; and all were supervised [15]. Each of these incentives contributed to the success of the intervention and increased motivation. In Burkina Faso, permission to retail treatment to patients meant that each transaction contributed to CHWs’ income. The cost was nominal for patients [16] but enabled CHWs to earn an average monthly income of USD $1.14 (maximum USD $3.61) during the peak 3-month malaria season and USD $.40 per month (maximum USD $1.28) during the rest of the year. These earnings, however, still fall short of the estimated value of time they contributed to healthcare.

The case for using CHWs depends on the assumption that there is a health benefit and that any alternative means of providing that benefit would cost more than using CHWs. We did not compare alternatives to improving access to healthcare, but the substantial health benefit provided in improving access and reducing illness duration with a modest CHW time allocation suggests that a cost per month that varies from USD $4.30 per person in Burkina Faso to USD $24.60 in Nigeria might be economically attractive.

The results from this article, in combination with the health outcomes results reported elsewhere [12], provide a strong case for the intervention to be scaled up at the cost of a modest increase in CHWs’ time. The key dilemma is how best to remunerate CHWs for their time and/or provide incentives that can sustain their contributions to healthcare. Financial remuneration may well be CHWs’ preferred option, but policies intent upon reducing their attrition rates and maintaining their effectiveness will need to respond to what motivates them. Sanou et al [15] indicate that a substantial component of CHWs’ motivation derives from their status in the community and technical supervision; these 2 components apparently reinforce their technical skills (and hopes of a career path) and link them to community members and the formal health system. If CHWs are highly motivated by community status, then policies can be designed to reinforce that status; if they are motivated more by income prospects, then compensation for their time needs to be structured in a way that improves income and efficient use of time (ie, reduces incentives to “waste time”). The model in Burkina Faso has the disadvantage of giving them an incentive to treat more, but a mixed model combining variations of the Burkina Faso approach plus nonfinancial incentives may be possible. Most CHWs in the study were relatively young with small children of their own, living in an agricultural area. Providing free health coverage for their children plus some financial motivation might be a model to be tested.

Nonfinancial incentives, such as training, adequate supervision, community recognition, certification, identification badges, reduced stockouts, and bicycles that are appropriate for the CHWs' work were mentioned by CHWs as helping to increase their sense of self-worth, a source of pride, and relevant to future job prospects [15]. They indicate that CHWs’ motivation, retention, and effectiveness is heavily influenced by who they are in the community context, how they are perceived by community members, and the extent to which they are supported to maintain a stable relationship with their clients. Appropriate training and supervision were stated to improve their confidence and competence, and regular replenishment of supplies ensured that their relationship with their clients was not undermined by stockouts. The most successful CHWs programs have recognized that it is important to use multiple nonfinancial incentives to build CHWs’ continuing sense of self-worth and consolidate their relationship with their communities—some pairing CHWs so that they can work together and provide mutual support, and others where the community provides free labor on the CHW's farm [17].

This study has several limitations. First, it may underestimate time allocated to healthcare, as some parents may have visited a CHW at night (between 6 pm and 6 am), but information during night visits was not collected. In Burkina Faso and, to a large degree, in Nigeria, data before intervention were collected during the peak of malaria transmission, whereas data during the intervention were collected more evenly. A comparison of time use before and during the intervention is therefore limited to the high malaria transmission season. In Nigeria, some CHWs were reluctant to fill in the time forms because this was perceived to increase their workload, whereas others enthusiastically completed multiple forms. To reduce the documentation burden and increase representativeness of time use across most CHWs, the research team assisted in completing the forms and we limited data use to 8 forms per CHW.

## Supplementary Data

Supplementary materials are available at http://cid.oxfordjournals.org. Consisting of data provided by the author to benefit the reader, the posted materials are not copyedited and are the sole responsibility of the author, so questions or comments should be addressed to the author.

Supplementary Data
