# Supplementary material for: Quantifying and Valuing Community Health Worker Time in Improving Access to Malaria Diagnosis and Treatment
Source: Clin Infect Dis. 2016 Dec 6;63(Suppl 5):S298–305. doi: 10.1093/cid/ciw629 (PMC5146701; doi:10.1093/cid/ciw629)
Supplement: Supplementary Data [file supp_63_suppl-5_S298__index.html]

Supplementary Data 

# Quantifying and Valuing Community Health Worker Time in Improving Access to Malaria Diagnosis and Treatment

## Supplementary Data

Supplementary Data

- Supplementary Data - Pdf file
