# Supplementary material for: Quantifying and Valuing Community Health Worker Time in Improving Access to Malaria Diagnosis and Treatment
Source: Clin Infect Dis. 2016 Dec 6;63(Suppl 5):S298–305. doi: 10.1093/cid/ciw629 (PMC5146701; doi:10.1093/cid/ciw629)
Supplement: Supplementary Data [file supp_ciw629_ciw629supp.pdf]

Supplementary Table 1. Allocation of community health worker's time across categories of daily activities during the intervention period stratified by low/high malaria season*

	DURING INTERVENTION											
	Burkina Faso (36 CHWs)				Nigeria (17 CHWs)				Uganda (147 CHWs)			
**Daily activity**	**Low season**		**High season**		**Low season**		**High season**		**Low season**		**High season**	
**Number of CHW's questionnaires**	**N=29**	**%**	**N=43**	**%**	**N=37**	**%**	**N=33**	**%**	**N=162**	**%**	**N=305**	**%**
**Healthcare provision**												
0 hour	26	90	5	12	2	5	1	3	8	5	18	6
>0-2 hours	3	10	30	70	31	84	30	91	150	93	261	86
>2 hours	-	-	8	19	4	11	2	6	4	2	26	8
Mean time (minutes, SD)	5.5 (18.3)	-	89.8 (63.0)	-	87.6 (81.7)	-	66.1 (36.6)	-	60.7 (28.8)	-	78.3 (65.1)	-
**Family / personal time and housework****												
0 hour	-	-	1	2	-	-	-	-	4	3	13	4
>0-4 hours	16	55	18	42	8	22	6	18	29	18	72	24
>4-8 hours	10	35	21	49	22	59	14	42	114	70	185	61
>8 hours	3	10	3	7	7	19	13	40	15	9	35	11
Mean time (minutes, SD)	264.1 (155.9)	-	247.9 (167.8)	-	366.5 (154.1)	-	427.3 (175.9)	-	350.9 (131.6)	-	326.2 (146.3)	-
**Agriculture**												
0 hour	2	7	5	12	15	41	13	40	6	4	29	10
>0-4 hours	6	21	9	21	14	38	8	24	67	41	119	39
>4-8 hours	10	34	19	44	6	16	11	33	84	52	147	48
>8 hours	11	38	10	23	2	5	1	3	5	3	10	3
Mean time (minutes, SD)	377.9 (201.4)	-	323.7 (189.3)	-	145.9 (168.3)	-	169.1 (177.8)	-	264.3 (108.2)	-	254.2 (128.2)	-
**Paid work**												
0 hour	29	100	42	98	34	92	32	97	162	100	302	99
>0-4 hours	-	-	-	-	1	23	1	3	-	-	1	0
>4 hours	-	-	1	2	2	5	-	-	-	-	2	1
Mean time (minutes, SD)	0.0 (0.0)	-	6.0 (39.6)	-	24.9 (87.0)	-	7.3 (41.8)	-	0.0 (0.0)	-	3.5 (39.7)	-
**Self employment**												
0 hour	24	82	37	86	19	51	26	79	151	93	260	85
>0-4 hours	1	4	3	7	16	43	7	21	1	1	20	7

>4-8 hours	3	10	2	5	2	6	-	-	7	4	18	6
>8 hours	1	4	1	2	-	-	-	-	3	2	7	2
Mean time (minutes, SD)	65.5 (158.5)	-	36.7 (107.2)	-	63.2 (101.3)	-	323.0 (47.5)	-	28.3 (112.6)	-	45.8 (130.0)	-
**Going to the market**												
0 hour	28	97	39	91	19	51	23	70	134	83	269	88
>0-2 hours	-	-	1	2	18	49	9	27	26	16	28	9
>2 hours	1	3	3	7	0	0	1	3	2	1	8	3
Mean time (minutes, SD)	5.5 (29.7)	-	14.0 (49.5)	-	31.4 (37.6)	-	26.7 (46.5)	-	15.8 (38.9)	-	11.8 (39.0)	-

Column percentages presented.

* High malaria season: Burkina Faso: July, August and September; Nigeria: January, May, June, July and August; Uganda: January, June, July, August, September and October.

** Includes taking care of their own children, cleaning the house, lighting a fire, cooking, fetching water, washing the clothes/dishes, taking a bath, eating, having lessons, praying/going to church, visiting someone, taking a nap.

Supplementary Table 2. Value of work time per season of CHWs during the intervention phase (based on the data of supplementary table 1)

	**Burkina Faso**			**Nigeria**			**Uganda**		
	**Low season**	**High season**	**Total for one year**	**Low season**	**High season**	**Total for one year**	**Low season**	**High season**	**Total for one year**
Estimated CHW time allocation to health per month (in hour, per CHW)	**2.8**	**44.9**	**13.3**	**43.8**	**33.1**	**39.3**	**30.4**	**39.2**	**34.8**
Estimated CHW time allocation to health for the season period/year (in hour, per CHW)	24.8	134.7	159.5	306.6	165.3	471.9	182.1	234.9	417.0
Minimum salary per month (in USD)*	**52.1**	**52.1**	**52.1**	**100.0**	**100.0**	**100.0**	**54.0**	**54.0**	**54.0**
Monetary value of time allocated to health per month per CHW (in USD)	0.9	14.6	4.3	27.4	20.7	24.6	10.2	13.2	11.7
Monetary value of time allocated to health for the season period/year per CHW (in USD)	8.1	43.9	51.9	191.6	103.3	294.9	61.5	79.3	140.7
Number of CHWs in the study districts**	**49**	**49**	**49**	**51**	**51**	**51**	**164**	**164**	**164**
Monetary value of time allocated to health for the season/year at study district level for all CHWs (in USD)***	395	2,149	2,544	9,773	5,267	15,040	10,079	13,002	23,081

* References [11, 13, 14].

** Burkina Faso: 50 CHWs in total but one was not accepted by the community.

*** for 2 districts in Uganda.

Supplementary Table 3. Mean CHW's income (in USD) from ACTs sold to sick children in Burkina Faso

	**During the intervention: Sept 2014 - Oct 2015**	**Estimate for 1 calendar year**	**High malaria season* during the intervention (3 months)**	**Low malaria season during the intervention (9 months)**
Number of CHWs**	49	49	49	49
Number of children < 37 months who received ACT	3,684	3,158	1,799	1,885
Number of children ≥37 months who received ACT	1,684	1,443	814	870
Total income in USD	343.68	294.58	167.09	176.59
Mean (SD) income per CHW in USD	7.01 (4.14)	6.01	3.41 (2.33)	3.60 (2.03)
Mean monthly income per CHW in USD	0.50	0.50	1.14 (max: 3.61)***	0.40 (max: 1.28)***

CHW, community health worker; ACT, artemisinin-based combination therapy.

* July, August & September.

** 50 CHWs in total but one was not accepted by the community.

*** The maximum is calculated based on number of patients treated with ACTs in Burkina Faso by CHWs (see Ajay et al. – Ref 1 in this supplement #83657).
